# Characterization of a Thioredoxin-1 Gene from *Taenia solium* and Its Encoding Product

**DOI:** 10.1155/2015/453469

**Published:** 2015-05-19

**Authors:** Lucía Jiménez, Oscar Rodríguez-Lima, Alicia Ochoa-Sánchez, Abraham Landa

**Affiliations:** Departamento de Microbiología y Parasitología, Facultad de Medicina, Universidad Nacional Autónoma de Mexico, Ciudad Universitaria, Edificio A, No. 2 Piso, 04510 Mexico, DF, Mexico

## Abstract

*Taenia solium* thioredoxin-1 gene (*TsTrx-1*) has a length of 771 bp with three exons and two introns. The core promoter gene presents two putative stress transcription factor binding sites, one putative TATA box, and a transcription start site (TSS). TsTrx-1 mRNA is expressed higher in larvae than in adult. This gene encodes a protein of 107 amino acids that presents the Trx active site (CGPC), the classical secondary structure of the thioredoxin fold, and the highest degree of identity with the *Echinococcus granulosus* Trx. A recombinant TsTrx-1 (rTsTrx-1) was produced in *Escherichia coli* with redox activity. Optimal activity for rTsTrx-1 was at pH 6.5 in the range of 15 to 25°C. The enzyme conserved activity for 3 h and lost it in 24 h at 37°C. rTsTrx-1 lost 50% activity after 1 h and lost activity completely in 24 h at temperatures higher than 55°C. Best storage temperature for rTsTrx-1 was at −70°C. It was inhibited by high concentrations of H_2_O_2_ and methylglyoxal (MG), but it was inhibited neither by NaCl nor by anti-rTsTrx-1 rabbit antibodies that strongly recognized a ~12 kDa band in extracts from several parasites. These TsTrx-1 properties open the opportunity to study its role in relationship *T. solium*-hosts.

## 1. Introduction

Thioredoxin (Trx) is a small (~12 kDa) enzyme that belongs to the reductase family. Trx reduces disulfides in several proteins using its conserved dithiol active site. It is ubiquitous and multifunctional; it is involved in processes such as maintenance of cellular homeostasis, cell proliferation, detoxification of peroxides (H_2_O_2_, hydroperoxides), DNA synthesis, signaling, and inhibition of apoptosis. Likewise it reduces diverse molecules of low molecular weights, such as glutathione disulfide, as well antioxidants dehydroascorbate, lipoic acid, and lipoamine [1–4]. All these events oxidize Trx, and it is reduced by thioredoxin glutathione reductase (TGR) and NADPH + H^+^; these components form the thioredoxin system in platyhelminths [5].

Trx has been classified into cytosolic (Trx-1) and mitochondrial (Trx-2); the latter is synthesized with an additional N-terminal extension that targets the mitochondrial protein, where it is cleaved to yield the ~12 kDa form [3]. All Trx enzymes have a similar structure, the Trx fold that is formed by a central domain with five-stranded *β*-sheet, surrounded by four *α*-helices, and the active site (CGPC), located between *β* strand 2 and *α*-helix 2 [3].

In Cestoda, Trx and TGR have been reported in* Echinococcus granulosus* and* Taenia crassiceps.* On the other hand, these organisms and* Taenia solium* possess a typical 2-Cys peroxiredoxin, which reduces H_2_O_2_ and hydroperoxides to water and its corresponding alcohol using the thioredoxin system. This shows that these organisms are able to regulate hydroperoxides levels and repair enzymes inactivated by oxidative stress [5–9].

Neurocysticercosis is the most common parasitic brain disease worldwide; moreover the high relationship between epilepsy and neurocysticercosis is considered now as a “biological marker” of the social and economic development of a community [10]. No commercial vaccine exists to prevent this parasitic disease and the treatment relies on two drugs, albendazole and praziquantel, to which* T. solium* has started to develop resistance [11, 12]. Therefore, the identification and biochemical characterization of new targets are important tools for development of vaccines or therapeutic drugs.

In this study, we describe the cloning and characterization of a gene that encodes a thioredoxin-1 from* Taenia solium* (*TsTrx-1*) and present a partial biochemical characterization of its encoding product.

## 2. Material and Methods

### 2.1. *Taenia solium Trx* Gene and cDNA Isolation

A Trx probe was generated by RT-PCR using the SuperScript One Step RT-PCR Kit (Invitrogen, Carlsbad, CA) with 1 *μ*g of* T. solium* larval total RNA prepared by TRIzol (Invitrogen, Carlsbad, CA) and two degenerated primers called TRX-1 and TRX-2 designed from the well conserved regions (TWCGPCK and MPTLFVFK) in Trx enzymes. The RT-PCR program for cDNA synthesis was 1 cycle at 50°C for 30 min, 30 cycles at 94°C for 1 min, 54°C for 30 sec, and 72°C for 1 min, and a final extension cycle at 72°C for 15 min. The fragment (probe) obtained was cloned into pCRII vector (Invitrogen), sequenced on an automated DNA sequencer ABI Prism model 373 (Perkin-Elmer, Applied Biosystem, Foster City, CA), and the nucleotide translation to amino acids sequence was analyzed with the PCGENE program. Screenings for* T. solium *cysticerci cDNA and genomic DNA libraries were carried out using 45,000 and 120,000 *λ*ZAPII phages, respectively. Both libraries were hybridized with the aforementioned probe, as previously described [8, 9]. Phage positive clones obtained after three screening rounds of each library were converted to Bluescript plasmids using ExAssist helper phage (Stratagene, La Jolla, CA). Plasmids were sequenced and analyzed as before. Intron detection was carried out with the PCGENE and analyses of amino acid sequences were performed through BLAST (National Center for Biotechnology Information NCBI (http://www.ncbi.nlm.nih.gov/BLAST/)). Alignment of the multiple amino acid sequences was performed by Clustal X (http://www.clustal.org/). The proximal promoter analysis for detecting putative transcription binding sites was carried out with the TRANSFAC program (http://www.gene-regulation.com/pub/databases.html).

### 2.2. Transcription Start Site Determination

*Taenia solium* larval total RNA (200 ng) was used as template for the transcription start site (TSS) determination using the Smart RACE cDNA Amplification Kit (Clontech Mountain View, CA). RACE fragments were amplified by PCR using reverse primer TRXRE-1 designed from the region DEMAKENAN (5′-GTTAGCATTCTCCTTTGCCATTTCGTC-3′) and forward primer SMARTII from kit (5′-AAGCAGTGGTATCAACGCAGAGTACGCGGG-3′) following manufacturer's directions. The resulting bands were cloned into pCRII (Invitrogen), sequenced, and compared with the results obtained with the neural network analysis tool (http://www.fruitfly.org/) to confirm transcription start site (TSS) found by the 5′-RACE method.

### 2.3. Transcripts Relative Expression

For the real time-PCR, 3 *μ*g of total RNA from* T. solium* larval and adult stages was reverse-transcribed to cDNA using SMARTScribe Reverse Transcriptase and 5′-CDS primer A (Clontech) according to manufacturer's instructions. cDNA 200 ng was used for each reaction in a volume of 10 *μ*L using the primers TRX-X1 and TRX-X2 designed from the regions (MSVEAVV) and (IQANV-) of* TsTrx-1*. Primers SOZ-2 and SOZ-6 were designed on the regions (KHGFHVH) and (GNAGGR-) of* T. solium* Cu/Zn superoxide dismutase (TsCu/ZnSOD) [13]. The reactions were performed with LightCycler 480 SYBR Green I Master in the LightCycler 480 System (Roche, Germany). The real time-PCR program used was 95°C for 10 min and then 40 cycles at 95°C for 15 sec and 52°C for 1 min and 72°C for 30 sec. The mRNA levels of* TsTrx-1* were normalized using the* TsCu/ZnSOD* as a housekeeping gene, and relative amounts of mRNA were calculated using the comparative CT method.

### 2.4. Purification of Recombinant TsTrx (rTsTrx-1)

Plasmid pRSET containing the cDNA coding region from TsTrx-1 was expressed on BL21(DE3) bacteria with 1 mM IPTG during 4 h. Bacteria were centrifuged at 10,000 ×g and the pellet was disrupted by sonication in a TrisED buffer (10 mM Tris, 1 mM EDTA, and 1 mM DTT, pH 7.5) plus 4 M urea. The supernatant was applied onto a Ni+ sepharose column (His Trap HP GE Healthcare) and eluted with TrisED plus urea using a linear gradient of imidazole (0, 50, 100, 200, 300, and 400 mM). Fractions containing high Trx activity were dialyzed in TrisED buffer and reloaded in the Ni+ sepharose column for a second purification process without urea. The Trx obtained was concentrated and proteins concentration was determined by the Lowry method. Purification process of rTsTrx-1 was visualized by 15% SDS-PAGE staining with Coomassie Brilliant Blue.

### 2.5. Production of Antibodies and Western Blot

A 10-week-old New Zealand rabbit was immunized subcutaneously with 100 *μ*g of the purified recombinant enzyme plus 10 *μ*g of saponin as adjuvant. Immunizations were conducted on days 1, 15, and 30. Antisera were obtained one week after the third immunization.

For western blot analysis, 1 *μ*g/mm of rTsTrx-1, rTrx-*E. coli*, and human T-cell recombinant Trx (rTrx-human, Sigma-Aldrich, St. Louis. MO) and likewise 5 *μ*g/mm of parasites crude extract and* T. solium* cysticerci excretion-secretion antigens (E/S Ag), prepared as described in [14], were separated by 15% SDS-PAGE and transferred onto a nitrocellulose membrane (Amersham Biosciences, Sweden). The membrane was blocked with 1% BSA in PBS containing 0.05% Tween 20 buffer and incubated for 2 h at room temperature with the anti-rTsTrx-1 serum (dilution 1 : 100). After 3 washes of 5 min with PBS containing 0.05% Tween, the membrane was incubated for 1 h with horseradish peroxidase- (HRP-) conjugated goat anti-rabbit antibody (1 : 2000). Bands recognized by the anti-rTsTrx-1 serum were visualized with 3,3′-diaminobenzidine (DAB) and H_2_O_2_ as substrate; normal rabbit serum was used as negative control at the same dilution.

### 2.6. Effect of pH, Temperature, Methylglyoxal (MG), H_2_O_2_, NaCl, and Anti-TsTrx-1 Antibodies on rTsTrx-1 Activity

The oxidoreductase activity of rTsTrx-1 was determined by the dithiothreitol (DTT)/insulin reduction method described by Holmgren [15]. Briefly thioredoxin reactions are coupled to DTT using insulin as the protein substrate. rTsTrx-1 or rTrx-*E. coli* at 1, 10, and 20 *μ*g were added to 160 *μ*M of insulin in PE buffer (100 mM potassium phosphate, pH 6.5, containing 2 mM EDTA and 1 mM DTT). Insulin reduction was monitored by measuring turbidity at 650 nm for 30 min in a spectrophotometer Ultrospec 3100 Pro (Amersham Biosciences). A sample without rTsTrx-1 was used as reference control.

Assays to determine the pH enzymatic stability were carried out with rTsTrx-1 dialyzed for 8 h in citrate buffer at pH of 3, 5, and 6, in PE buffer at pH of 6.5, 7.5, and in Tris buffer at pH of 8, 9, and 10. The thermal stability of the enzyme was assayed incubating rTsTrx-1 at temperatures between 15 and 100°C during 1, 3, and 24 h. In both assays the Trx residual activity was measured. In addition, the optimal storage temperature was analyzed by incubating the enzyme at temperatures ranging from 25° to −70°C during 1 to 28 days.

To determine whether concentrations of 0 to 8 mM of MG, 0 to 2 M of NaCl, 0 to 200 mM H_2_O_2_, and 1, 10, and 20 *μ*g of IgG fraction, coming from sera of rabbit immunized with rTsTrx-1 and normal rabbit serum (control), affected the TsTrx-1 activity, the enzyme was incubated in each one for 30 min at 37°C and the activity was measured as before. For all these assays 20 *μ*g of enzyme was used.

## 3. Results

### 3.1. Isolation of cDNA and Gene Encoding* TsTrx-1* and Its Characterization

**Figure 1:**
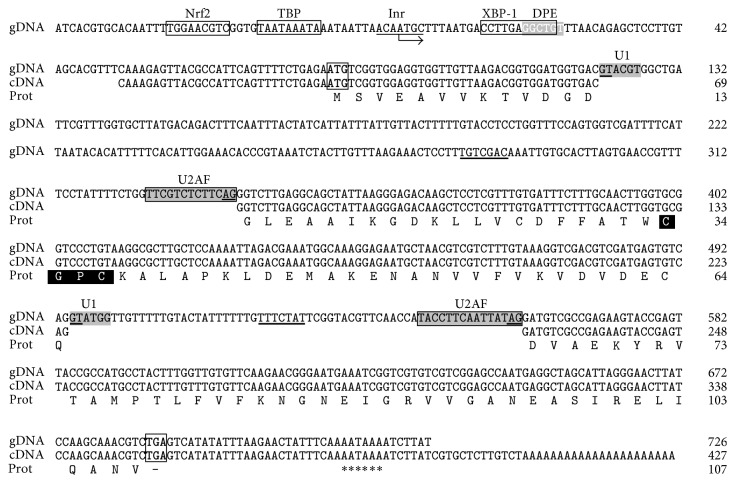
Genomic (gDNA) and complementary DNA (cDNA) nucleotides sequences and deduced protein from* Taenia solium* thioredoxin-1. Putative transcription factors sites (Nrf2, TBP, and XBP-1) are placed inside a box; DPE is in a white letter inside the grey box; TSS inside the Inr is underlined and signalized by an arrow. Start (ATG) and stop (TGA) codons are inside the box; donor (GT) and acceptor (AG) introns sequences are underlined. Putative branch point is underlined by a black bar; putative U1 and U2AF splicing binding sites are in grey inside a box; polyadenylation sites are indicated by asterisks (∗). Thioredoxin residues from the active site (CGPC) are highlighted in white on a black background.

Through RT-PCR, using total RNA from larval* T. solium* and primers designed on two conserved regions from several Trxs, we obtained a ~153 bp DNA fragment that evidenced high homology with* Trx* genes. This was used as probe to isolate the transcript and the coding* TsTrx-1* gene by screening a genomic DNA and cDNA *λ*ZAPII libraries, respectively.  Figure 1 shows the isolate genomic DNA sequence; it spans 771 bp and codes for the same Trx-1 as the cDNA. The 5′-RACE experiments on the proximal promoter of the* TsTrx-1* gene showed that the transcription start site (TSS) corresponds to an adenine (A) located within the initiator (Inr, ACAATGC) sequence and mapped at 81 bp upstream of the translation start codon (ATG). Moreover, we identified a putative TATA box located at −19 pb and a GGCTGT motif (downstream promoter element, DPE) at +22 pb, both from the TSS. Additionally, putative binding sites for Nrf2 and XBP1 transcription factors were found at −32 bp and +14 bp, respectively (GenBank accession for* TsTrx-1* gene is KM401604).

The structural coding region for the* TsTrx-1* gene spans over 770 pb. It has three exons split by two introns (intron I: 218 bp length; intron II: 65 bp length) that possess the donor-acceptor sites (NGT-AGN). Moreover, the putative binding sites for the splicing machinery U1 (intron I: ^121^ **GT**ACGT^126^; intron II: ^495^ **GT**ATGG^500^) and U2AF (intron I: ^326^TTCGTCTCTTC**AG** ^338^; intron II: ^547^CCTTCAATTAT**AG** ^459^) were identified. Additionally, the putative branching point in each intron (intron I: ^281^TGTCGAC^287^; intron II: ^522^TTTCTAT^528^) was identified too.  Figure 1 also shows the isolated cDNA with 427 bp with an open reading frame (ORF) from 31 (ATG) to 354 (TGA) bp that encodes for a protein with 107 amino acids with a theoretical molecular weight of 11,579 Da and pI of 4.39. It presents the motif (CGPC) that corresponds to the catalytic active site of Trx enzymes. Furthermore, a putative classic polyadenylation (AATAAA) site was located between 380 and 385 bp downstream of the stop codon (GenBank accession for TsTrx-1 cDNA is KM401605).

**Figure 2:**
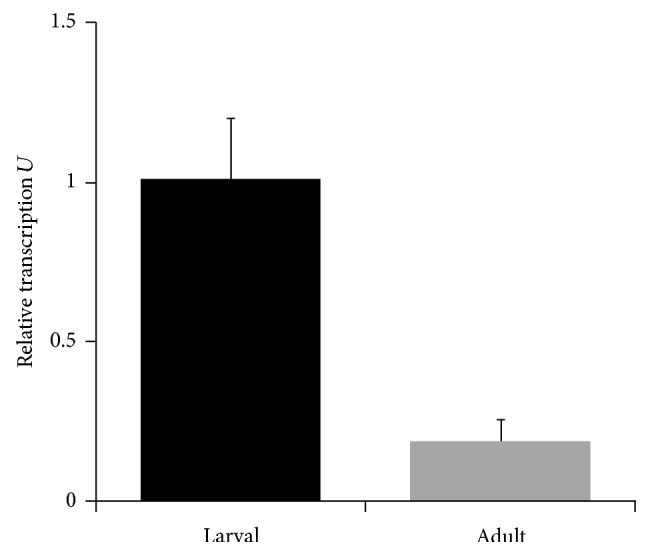
Relative transcription of* T. solium* thioredoxin-1 (*TsTrx-1*) gene from larvae and adult stages of* T. solium* was done by real time-PCR using TRX-X1 and TRX-X2 primers.

Table 1 depicts a comparison of the coding structural region of the* TsTrx-1* gene with* Trx-1* genes from* E. granulosus*,* Schistosoma mansoni*, human, and mouse [16–19]. It shows that the* Trx-1* genes of* T. solium*,* E. granulosus*, and* S. mansoni* have a similar size, in contrast to mammalian* Trx-1* genes that are bigger. It also depicts that* T. solium* and* E. granulosus* present an identical structural coding region to that of the* Trx-1* gene composed by three exons and two introns. Similarly,* S. mansoni Trx-1* gene also presents three exons and two introns, which are slightly larger than* T. solium* and* E. granulosus Trx-1* genes. In contrast, human and mouse genes present 5 exons and 4 introns with similar sizes between them, but introns are larger than those of cestodes. It is noteworthy that the second introns of* TsTrx-1 *and* E. granulosus* genes coincide with the second intron of* S. mansoni* and third intron of human and mouse* Trx-1* genes.  Figure 2 shows that TsTrx-1 mRNA was expressed higher in larvae than in adult, as determined by real time-PCR assays.

**Figure 3:**
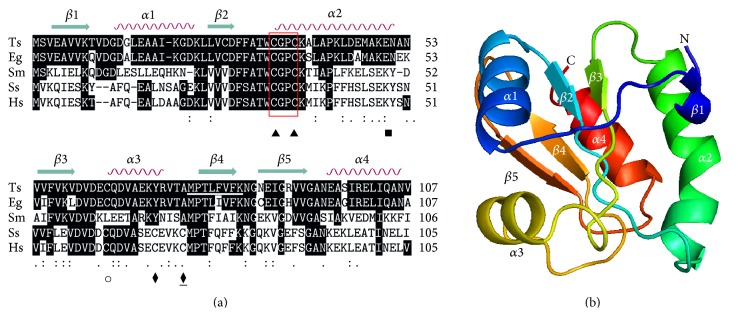
(a) Alignment of Trx-1 from* Taenia solium* (Ts. GenBank accession KM401605) with other thioredoxins from* Echinococcus granulosus* (Eg. GenBank: AF034637.1),* Schistosoma mansoni* (Sm. GenBank: AAL79841.1),* Sus scrofa* (Ss. GenBank NM_214313.2), and* Homo sapiens* (Hs. GenBank AF085844.1). Identical residues are highlighted in white on a black background. The symbols in the residues indicate (-) absence and (**:**) homology. Tyrosine 49 where nitration occurs (■) in mammalian. Cysteines: (▲) from active site, (○) conserved cysteine in mammalian and helminths, and (⧫) only present in mammalians where S-nitrosylation occurs; likewise (⧫) it is involved in glutathionylation. In a box is the active site and underlined are the residues used for primer design to produce the Trx-1 probe. Secondary structure elements are shown above the alignment. (b) Structure model of TsTrx-1. It shows the Trx fold formed by a central domain with five-stranded *β*-sheet, surrounded by four *α*-helices. The model was drawn with the Swiss Model program (http://swissmodel.expasy.org/).

The comparison of the deduced amino acid sequence of TsTrx-1 with* E. granulosus* Trx revealed (Figure 3(a)) an 87.85% identity, followed by a 46.72% with* S. mansoni*. In contrast, a low identity, between 41.12 and 43.92%, was found with pig and human Trx-1, respectively. TsTrx-1 has the two conserved cysteines (Cys34 and Cys37) in its active site and cysteine 64, which is conserved in cestodes and mammalian, but it is not presented in* S. mansoni* Trx-1. Cysteine 27 is shared only by* T. solium* and* E. granulosus* Trx-1; unfortunately, its function is still unknown.* T. solium* and* E. granulosus* Trx-1 as well as* S. mansoni* lack tyrosine 49 and cysteines 69 and 73, present in mammalians, which are involved in nitration, glutathionylation, and S-nitrosylation and dimer formation [20].  Figure 3(b) shows a model constituted by a central domain with five-stranded *β*-sheet, surrounded by four *α*-helices, showing the classical Trx fold and the active site (CGPC) located between *β* strand 2 and *α*-helix 2 [3].

### 3.2. Production and Characterization of rTsTrx-1

**Figure 4:**
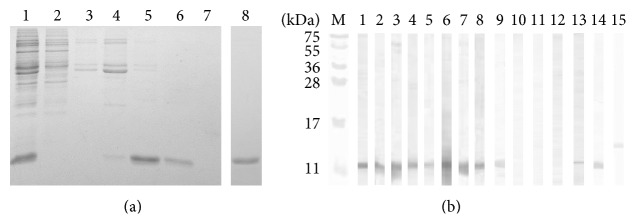
Purification process of the recombinant* T. solium* thioredoxin-1 (rTsTrx-1) and specificity of rabbit anti-TsTrx-1 serum. (a) 15% SDS-PAGE showing the crude extract of* Escherichia coli* produced with 4 M urea induced with IPTG, 1: after and 2: before. Crude extract was run through the nickel chelator column; 3: wash column. Eluted fractions with imidazole at 4: 50 mM, 5: 100 mM, 6: 200 mM, and 7: 400 mM. Eluted fractions from 100 and 200 mM were mixed and dialyzed and run through the same column to obtain 8: a pure rTsTrx-1. (b) Western blot showing the reaction from anti-TsTrx-1 serum with 1: pure rTsTrx-1 and crude extracts from 2:* Taenia solium *larvae, 3:* T. solium *adult, 4:* T. saginata *adult, 5:* T. taeniaeformis *adult, 6:* T. crassiceps* larvae, 7:* Hymenolepis diminuta *adult, 8:* Fasciola hepatica *adult, 9:* Entamoeba histolytica*, 10:* E. coli*, and 11:* Homo sapiens *recombinant Trxs. 12: a preimmune serum was incubated with a crude extract of* T. solium *larvae as a negative control. 13:* T. solium* cysticerci E/S Ag. Strips 14 and 15 show the rTrx-*E. coli* and Trx-*Homo sapiens* stained with Ponceau red. Molecular mass standards are indicated in the middle of both figures.

*Escherichia coli* containing the expression vector pRSETB with the coding region for TsTrx-1 were induced with IPTG for 4 h; bacteria were centrifuged and the pellet was disrupted with 4 M urea. Because rTsTrx-1 was produced with six histidines in the amino terminal, the supernatant was through to a nickel affinity chromatography.  Figure 4(a) shows the expression levels of the recombinant enzyme in* E. coli* and the purification steps were run on a 15% reduced SDS-PAGE. Lane 1 shows all the soluble proteins from* E. coli* induced with IPTG (a large band ~12 kDa is highlighted in the sample), whereas lane 2 presents the soluble proteins from* E. coli* before induction with IPTG. Lane 3 shows the wash fraction before the elution step. Imidazole fractions were showed at lanes 4 to 7 (50, 100, 200, and 400 mM); rTsTrx-1 was eluted in the 100 to 200 mM imidazole. These fractions were pooled and loaded again on the same nickel affinity chromatography, following the same procedure without urea. A single band with an apparent* Mr* of 12 kDa (rTsTrx-1) was obtained in fractions with 100 to 200 mM of imidazole (lane 8), pooled fractions were dialyzed in TrisED buffer, and protein concentration was determined. The entire process yielded 10 mg/L of culture medium.  Figure 4(b) shows the strong recognition of a band of ~12 kDa by the specific anti-TsTrx-1 antibodies in the western blot membranes containing the purified rTsTrx-1 (lane 1), crude extracts from larvae (lane 2), and adult (lane 3)* T. solium* stages and crude extracts from adult* T. saginata* (lane 4), adult* T. taeniaeformis* (lane 5), larval* T. crassiceps* (lane 6), adult* Hymenolepis diminuta* (lane 7), adult* Fasciola hepatica* (lane 8), and a weakly recognition for* Entamoeba histolytica* (lane 9). Anti-TsTrx-1 antibodies were not recognized in* E. coli *(lane 10) and* Homo sapiens *(lane 11) Trxs. The preimmune serum obtained from rabbit before immunization (lane 12) also did not recognize any band. Additionally, anti-TsTrx-1 antibodies strongly recognized a ~12 kDa band in cysticerci* T. solium* E/S Ag. Strips 14 and 15 are* E. coli* and* Homo sapiens* Trxs stained with Ponceau red.

### 3.3. Enzyme Activity and Optimal pH

**Figure 5:**
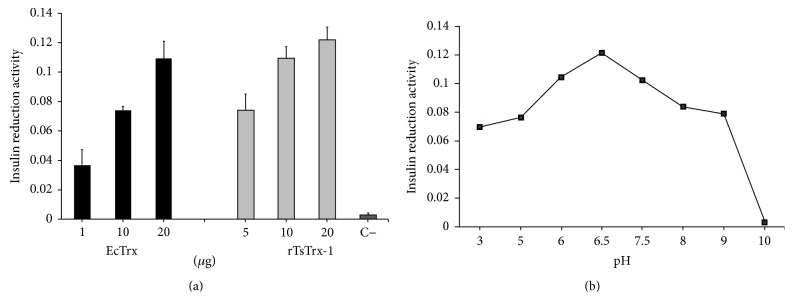
(a) Thioredoxin-catalyzed reduction of insulin. The increase in turbidity at 650 nm is plotted against the 1, 10, and 20 *μ*g of* E. coli* rTrx (dark bars) and 5, 10, and 20 *μ*g of* Taenia solium* thioredoxin-1 (rTsTrx-1, grey bars). (C−) Control lacking rTsTrx-1. (b) The pH enzymatic stability was determined incubating 20 *μ*g of rTsTrx-1 at different pH between 3 and 10. The residual activity was measured as before.

Figure 5(a) shows the insulin reduction activity performed with concentrations of 1 to 20 *μ*g of rTsTrx-1 at room temperature; all showed detectable insulin precipitation, but velocity and quantity of insulin reduction were dependent on rTsTrx-1 concentration. The maximal rate of precipitation was obtained with 20 *μ*g (1.5 *μ*M); however, there are no significant differences between 10 and 20 *μ*g; even 5 *μ*g reduced by half the amount of insulin precipitated. Therefore, the 20 *μ*g concentration was used for the characterization assays. Similar results were obtained with rTrx-*E. coli* (control assay) which showed a better activity at 20 *μ*g. A control reaction without rTsTrx-1 enzyme was carried out in parallel.  Figure 5(b) depicts the enzyme exhibiting a triangle-shaped curve showing high activity (more than 84%) over a broad range of pH, between 6 and 7.5, but the optimal pH was 6.5 in PE buffer. The same figure shows that the activity was maintained around 58% at pH of 3, 5, 8, and 9 and was lost completely at pH 10.

### 3.4. Temperature Effect

**Figure 6:**
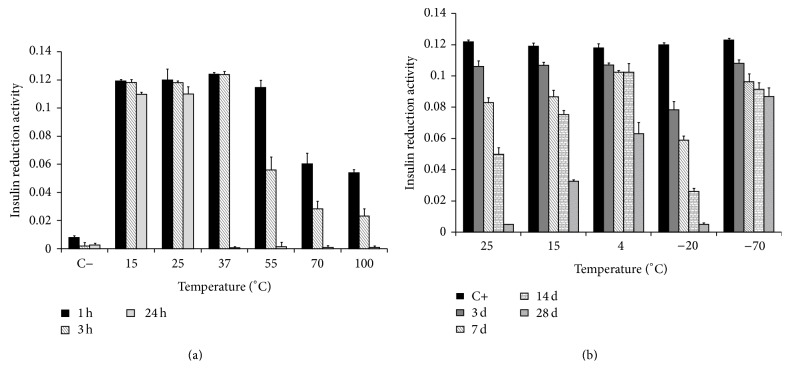
Effect of temperature on* T. solium* thioredoxin-1 (rTsTrx-1) activity. (a) rTsTrx-1 was incubated for 1, 3, and 24 hours at 15°C, 25°C, 37°C, 55°C, 70°C, and 100°C. (C−) Control lacking rTsTrx-1. (b) rTsTrx-1 was incubated during 3 to 28 days at 25°C, 15°C, 4°C, −20°C, and −70°C. (C+) Control of enzymatic activity was performed with a freshly made rTsTrx-1. Residual activity was determined by reduction of insulin assay.

Figure 6(a) shows the residual activity after exposing the rTsTrx-1 to different temperatures (15 to 100°C) at times (1, 3, and 24 h). The activity/temperature plots show a descending pattern. The enzyme maintained 100% activity for 3 h and lost it in 24 h at 37°C; at 55°C, 100% activity was maintained for 1 h; ~50% activity was lost at 3 h and lost activity completetly in 24 h. Finally at 70 and 100°C, the enzyme lost ~50%, ~75%, and 100% of activity at 1, 3, and 24 h, respectively. As negatives controls, we used a similar reaction without the Trx enzyme at all times; as expected, there was no activity. Noteworthy is that the loss of activity was not reversible in any condition.

To determine the best temperature to store the rTsTrx-1, assays such as those showed in Figure 6(b) were done. It shows that the enzyme stored at 25°C gradually lost 10%, 30%, and 60% of activity at 3, 7, and 14 days, respectively; at this same temperature, the enzyme lost the activity completely at 28 days. Decrease in activity at 15°C was of 10, 33, 42, and 70% at the tested times. Storage at 4°C induced a ~10% activity loss at 14 days and activity loss of 50% at day 28, whereas storage at 20°C induced a gradual activity loss of 33%, 50%, 85%, and almost 100% between days 3 and 28; at −70°C, the enzyme presented the best stability; it lost only 9% activity at 3 days and ~25% of activity between days 7 and 28.

### 3.5. Effect of NaCl, MG, H_2_O_2_, and Anti-TsTrx-1 Antibodies on rTsTrx-1

**Figure 7:**
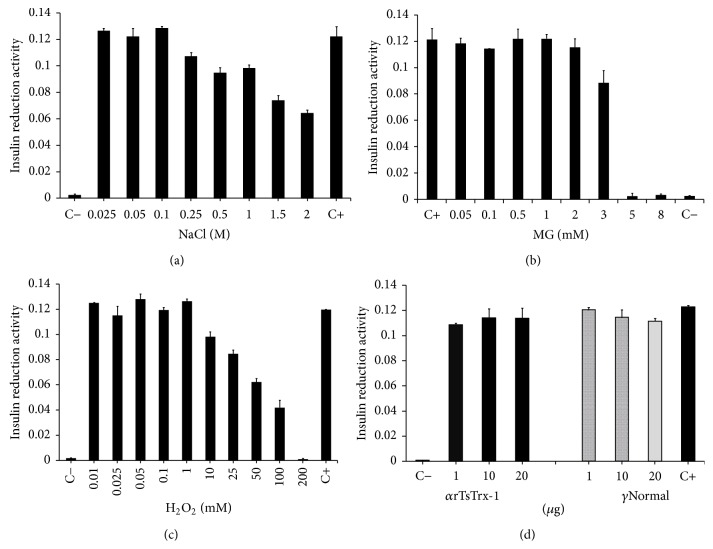
Effect on* T. solium* thioredoxin-1 (TsTrx-1) after incubation with different concentrations of (a) NaCl (25–2 M), (b) methylglyoxal (MG, 0.05–8 mM), (c) H_2_O_2_ (10 *μ*M–200 mM), and (d) IgG rabbit anti-*T. solium* Trx-1 and IgG from preimmune serum (1, 10, and 20 *μ*g). (C−) Control lacking rTsTrx-1 and (C+) control of enzymatic activity was performed with a freshly made rTsTrx-1.

Exposure of rTsTrx-1 at 37°C for 30 min to different NaCl concentrations showed that concentrations of 250, 500, 1000, 1500, and 2000 mM decreased its activity ~9, 17, 17, 40, and 50%, respectively (Figure 7(a)). Moreover, exposure of rTsTrx-1 at 3 mM MG decreased 25% of its activity, and higher concentrations inactivated rTsTrx-1 activity completely (Figure 7(b)). Concentration until 1 mM of H_2_O_2_ did not affect the enzyme activity of rTsTrx-1, whereas concentrations between 10 mM and 100 mM of H_2_O_2_ reduced the enzymatic activity (17, 25, 50, and 60%); concentration of 200 mM completely disrupted the activity (Figure 7(c)). A reaction without enzyme (negative control, C−) and a reaction with 1.5 *μ*M rTsTrx-1 without treatment (positive control, C+) were used as controls. As expected, no activity was detected in C−; in contrast 100% activity was obtained in C+. Finally, anti-TsTrx-1 antibodies were incapable of inhibiting enzymatic activity (Figure 7(d)) and, as expected, normal IgG did not affect the TsTrx-1 activity.

## 4. Discussion

The analysis of the 5′-flanking region of the* TsTrx-1* gene reveals putative sites for Nrf2 and XBP1 transcription factors; these sites are presented in promoters for typical 2-Cys-peroxiredoxin of* T. solium *and* Trx-1* from human genes and both factors are positive regulators of antioxidant genes under stress condition [9, 16, 21–23]. This suggests* Trx-1 *gene in cestodes could be regulated in this way. On the other hand, a putative TATA box was found at −19 bp and a DPE putative site at +22 bp, both related to TSS. It is known that the TATA box usually appears at −30 bp and DPE at +28 to +32 bp, even in Taeniidae family genes [9, 24, 25], and, for these reasons, neither TATA box nor DPE in the* TsTrx-1* gene is at a classical distance to be functional [26]. However, these assertions must be corroborated with functional studies. The comparison analysis of Inr sequences from the* TsTrx-1* gene with other Inr sequences from genes of the Taeniidae family suggests conservation in this motif with similarities to the mammalian consensus Inr sequence YYANWYY [9, 13, 24–26].

Comparison analyses of the coding region for the* Trx-1* gene show that* T. solium *(0.605 kb) and* E. granulosus* (0.609 kb) were identical. Even* S. mansoni Trx-1* gene was showed to have similar structure with three exons and two small introns; the composition of nucleotides and amino acids sequences is different. In contrast, they are different from the structure of human (~17 kb) and mouse (13 kb) genes, which have five exons and four big introns. These differences between number and sequence of introns could be used to design specific primers for the diagnosis of cysticercosis/taeniasis caused by* T. solium* using PCR with cerebrospinal fluid (CSF) and human feces [27]. This point is important for epidemiological studies, because it would allow identifying active infection of carriers of this parasite, which will be easy to treat with antihelminthic drugs. Moreover, the difference in introns and the presence of an intron in the same position in different* Trx-1* genes of different organisms suggest that it was present in the ancestor; however more detailed studies should be done to use intron position as marker of evolution [28].

The confrontation of cysticerci with the host immune response (inflammation) and oxidative stress in tissues with high oxygen, such as brain and muscle, in contrast to the adult stage that lives in the small intestine, where it is exposed less to these factors, could be the reason why RNA expression of the* TsTrx-1* gene is higher in cysticerci than in adults.

The primary sequence shows a typical catalytic site (CGPC), which executes the oxidoreductase activity, and the typical thioredoxin fold; even more, it shows higher identity with Trx from* E. granulosus*, a cestode, less identity with* S. mansoni*, a trematode, and poor identity with pigs and humans (intermediate and accidental hosts of* T. solium*). Polyclonal antibodies produced against rTsTrx-1 strongly recognized a ~12 kDa band in various* Taenia* species and* F. hepatica* and weakly in* E. histolytica* but did not recognize* E. coli* and human Trxs. These points suggest that specific regions of TsTrx-1 could be used in vaccination assays against* T. solium*.

Noteworthy, TsTrx-1,* E. granulosus*, and* S. mansoni* Trx-1 have a cysteine at position 27 close to the cysteines of the active site; moreover they lack cysteines 69 and 73, which regulate the activity and biological functions of the mammalian Trx-1. These findings give rise to the following questions. Does cysteine 27 have a role in the catalytical activity? Are the helminths Trx-1 not regulated by posttranscriptional modifications, and which is the biological consequence of this? [20].

The rTsTrx-1 enzyme exerted its optimal reductase activity in a range of pH and temperature of 6.5 to 7.5 and 4 to 37°C, respectively. Likewise, it presented 100% activity in a buffer with 100 mM NaCl and lost it gradually in a buffer with concentrations higher than 250 mM NaCl, losing up to 50% activity at 2 mM NaCl. On the other hand, the best way to store this enzyme to not lose its activity was at −70°C for 28 days and at 4°C for 14 days. The known biochemical properties of the enzyme, such as pH, temperature, buffers, cofactors, salt concentration, additives as glycerol, and oxidants and inhibitors, let us preserve its activity, which will help to determine enzymatic mechanisms with inhibitors or observe its effect on cells or organisms* in vitro* and* in vivo*.

The presence of MG and ROS especially H_2_O_2_ triggers oxidative stress, DNA damage, and apoptosis in cells. MG is a reactive carbonyl compound that causes glycation of proteins and is formed principally by glucose metabolism; it induces oxidative stress by inactivating antioxidant enzymes, such as Cu/Zn superoxide dismutase, glutathione peroxidase, and Trx reductase and decreases Trx protein level [29]. Activity of rTsTrx-1 was not affected by 2 mM of MG. On the other hand, ROS are continuously produced by the host's inflammation caused by the immune response. It is known that helminths lack catalase and present low activity of glutathione peroxidase; cysticerci of* T. crassiceps* (cestode) resist concentration of 2.5 mM of H_2_O_2_ *in vitro* for 2.5 h. We observed that rTsTrx-1 enzyme resists 1 mM H_2_O_2_. Both findings indicate that rTsTrx-1 is highly resistant to oxidant molecules and, together with the 2-Cys-peroxiredoxins of* T. solium*, could constitute the hydroperoxides-regulating system in this parasite [8, 9].

Trx is an essential component of the thioredoxin system, where it performs functions such as antioxidative, protein-reducing, and signal-transducing ones. In mammalian and helminths, the antioxidative activity is the most studied. However, there is evidence that Trx participates in signaling pathways, interacting with different proteins, to control processes such as development, proliferation, migration, apoptosis, inflammation, and metabolism [30]. No signal sequence was found on the* TsTrx-1* gene; however Trx-1 was found in the cysticerci E/S Ag and several reports have observed that mammalian cells stimulated with lipopolysaccharide and viral infections are able to secrete Trx-1 [31, 32]. In addition, now it is known that helminths constitutively secrete Trx-1 and molecules that are able to modify the immune response by altering the normal signaling of host immune cells, letting them drive Th2 immune response, which allows for their long term establishment, such as the cases of 2-Cys-peroxiredoxins from* S. mansoni* and* F. hepatica* [33, 34].

In conclusion, the Trx-1 tools presented here could help to perform studies to know the role that Trx-1 plays in the host-parasite relationship; likewise its antioxidant and biochemical properties could be used to inactivate TsTrx-1 by drug or by vaccine. Furthermore, its importance in immune signaling pathways could let us think of it as a therapeutical molecule to other diseases.

## Figures and Tables

**Table 1:** Comparison of the structural coding regions of *Taenia solium Trx-1* gene (*TsTrx-1*) with other *Trx-1* genes from *Homo sapiens*, *Mus musculus*, *Schistosoma mansoni*, and *Echinococcus granulosus*.

Organism	Size of structural coding region of gene (kbp)	Number of introns, size (kbp), and position	Number and size of exons (bp)	Size encoding product (amino acids)
*Taenia solium *	0.605	I: 0.218 (DGD^13^–^14^GLE) II: 0.065 (ECQ^65^–^66^DVA)	1: 39 2: 156 3: 129	107

*Echinococcus granulosus *	0.609	I: 0.220 (DGD^13^–^14^ALE) II: 0.065 (ECQ^65^–^66^DVA)	1: 39 2: 156 3: 129	107

*Schistosoma mansoni *	0.685	I: 0.237 (KQD^10^–^11^GDL) II: 0.126 (KLE^64^–^65^ETA)	1: 30 2: 162 3: 129	106

*Mus musculus *	~13	I: 4.2 (ESK^8^–^9^EAF) II: 0.897 (FFH^43^–^44^SLC) III: 5.6 (DCQ^63^–^64^DVA) IV: 1.1 (GQK^85^–^86^VGE)	1: 24 2: 105 3: 60 4: 66 5: 63	105

*Homo sapiens *	~17	I: 5.0 (ESK^8^–^9^TAF) II: 0.478 (FFH^43^–^44^SLS) III: 5.9 (DCQ^63^–^64^DVA) IV: 0.558 (GQK^85^–^86^VGE)	1: 24 2: 105 3: 60 4: 66 5: 63	105

## References

[1] Powis G., Montfort W. R. (2001). Properties and biological activities of thioredoxins. *Annual Review of Pharmacology and Toxicology*.

[2] Chae H. Z., Robison K., Poole L. B., Church G., Storz G., Rhee S. G. (1994). Cloning and sequencing of thiol-specific antioxidant from mammalian brain: alkyl hydroperoxide reductase and thiol-specific antioxidant define a large family of antioxidant enzymes. *Proceedings of the National Academy of Sciences of the United States of America*.

[3] Arnér E. S. J., Holmgren A. (2000). Physiological functions of thioredoxin and thioredoxin reductase. *European Journal of Biochemistry*.

[4] Jortzik E., Becker K. (2012). Thioredoxin and glutathione systems in *Plasmodium falciparum*. *International Journal of Medical Microbiology*.

[5] Salinas G., Selkirk M. E., Chalar C., Maizels R. M., Fernández C. (2004). Linked thioredoxin-glutathione systems in platyhelminths. *Trends in Parasitology*.

[6] Chalar C., Martínez C., Agorio A., Salinas G., Soto J., Ehrlich R. (1999). Molecular cloning and characterization of a thioredoxin gene from *Echinococcus granulosus*. *Biochemical and Biophysical Research Communications*.

[7] Rendón J. L., del Arenal I. P., Guevara-Flores A., Uribe A., Plancarte A., Mendoza-Hernández G. (2004). Purification, characterization and kinetic properties of the multifunctional thioredoxin-glutathione reductase from *Taenia crassiceps* metacestode (cysticerci). *Molecular and Biochemical Parasitology*.

[8] Molina-López J., Jiménez L., Ochoa-Sánchez A., Landa A. (2006). Molecular cloning and characterization of a 2-Cys peroxiredoxin from *Taenia solium*. *Journal of Parasitology*.

[9] Vaca-Paniagua F., Parra-Unda R., Landa A. (2009). Characterization of one typical 2-Cys Peroxiredoxin gene of *Taenia solium* and *Taenia crassiceps*. *Parasitology Research*.

[10] Mewara A., Goyal K., Sehgal R. (2013). Neurocysticercosis: a disease of neglect. *Tropical Parasitology*.

[11] Chong M. S., Hawkins C. P., Cook G. C., Hawkes C. H., Kocen R. S. (1991). A resistant case of neurocystercercosis. *Postgraduate Medical Journal*.

[12] Hasan M. S., Bin Basri H., Hin L. P., Stanslas J. (2011). Surgical remotion of a cysticercotic granuloma responsible for refractory seizures: a case report. *Surgical Neurology International*.

[13] Parra-Unda R., Vaca-Paniagua F., Jiménez L., Landa A. (2012). Cu, Zn superoxide dismutase: cloning and analysis of the *Taenia solium* gene and *Taenia crassiceps* cDNA. *Experimental Parasitology*.

[14] Ochoa-Sánchez A., Jiménez L., Landa A. (2011). The hamster model for identification of specific antigens of *Taenia solium* tapeworms. *Journal of Biomedicine and Biotechnology*.

[15] Holmgren A. (1979). Thioredoxin catalyzes the reduction of insulin disulfides by dithiothreitol and dihydrolipoamide. *The Journal of Biological Chemistry*.

[16] Kaghad M., Dessarps F., Jacquemin-Sablon H., Caput D., Fradelizi D., Wollman E. E. (1994). Genomic cloning of human thioredoxin-encoding gene: mapping of the transcription start point and analysis of the promoter. *Gene*.

[17] Church D. M., Goodstadt L., Hillier L. W. (2009). Lineage-specific biology revealed by a finished genome assembly of the mouse. *PLoS Biology*.

[18] Protasio A. V., Tsai I. J., Babbage A. (2012). A systematically improved high quality genome and transcriptome of the human blood fluke *Schistosoma mansoni*. *PLoS Neglected Tropical Diseases*.

[19] Tsai I. J., Zarowiecki M., Holroyd N. (2013). The genomes of four tapeworm species reveal adaptations to parasitism. *Nature*.

[20] Haendeler J. (2006). Thioredoxin-1 and posttranslational modifications. *Antioxidants & Redox Signaling*.

[21] Mai B., Breeden L. (1997). Xbp1, a stress-induced transcriptional repressor of the *Saccharomyces cerevisiae* Swi4/Mbp1 family. *Molecular and Cellular Biology*.

[22] Ma Q. (2013). Role of Nrf2 in oxidative stress and toxicity. *Annual Review of Pharmacology and Toxicology*.

[23] Hawkes H.-J. K., Karlenius T. C., Tonissen K. F. (2014). Regulation of the human thioredoxin gene promoter and its key substrates: a study of functional and putative regulatory elements. *Biochimica et Biophysica Acta*.

[24] Campos A., Bernard P., Fauconnier A. (1990). Cloning and sequencing of two actin genes from *Taenia solium* (Cestoda). *Molecular and Biochemical Parasitology*.

[25] da Silva C. M. D., Ferreira H. B., Picón M., Gorfinkiel N., Ehrlich R., Zaha A. (1993). Molecular cloning and characterization of actin genes from *Echinococcus granulosus*. *Molecular and Biochemical Parasitology*.

[26] Juven-Gershon T., Hsu J.-Y., Theisen J. W., Kadonaga J. T. (2008). The RNA polymerase II core promoter—the gateway to transcription. *Current Opinion in Cell Biology*.

[27] Rodriguez S., Wilkins P., Dorny P. (2012). Immunological and molecular diagnosis of cysticercosis. *Pathogens and Global Health*.

[28] Sahrawy M., Hecht V., Lopez-Jaramillo J., Chueca A., Chartier Y., Meyer Y. (1996). Intron position as an evolutionary marker of thioredoxins and thioredoxin domains. *Journal of Molecular Evolution*.

[29] Tatsunami R., Oba T., Takahashi K., Tampo Y. (2009). Methylglyoxal causes dysfunction of thioredoxin and thioredoxin reductase in endothelial cells. *Journal of Pharmacological Sciences*.

[30] Lee S., Kim S. M., Lee R. T. (2013). Thioredoxin and thioredoxin target proteins: from molecular mechanisms to functional significance. *Antioxidants & Redox Signaling*.

[31] Ejima K., Koji T., Nanri H., Kashimura M., Ikeda M. (1999). Expression of thioredoxin and thioredoxin reductase in placentae of pregnant mice exposed to lipopolysaccharide. *Placenta*.

[32] Sahaf B., Rosén A. (2000). Secretion of 10-kDa and 12-kDa thioredoxin species from blood monocytes and transformed leukocytes. *Antioxidants and Redox Signaling*.

[33] Donnelly S., Stack C. M., O'Neill S. M., Sayed A. A., Williams D. L., Dalton J. P. (2008). Helminth 2-Cys peroxiredoxin drives Th2 responses through a mechanism involving alternatively activated macrophages. *FASEB Journal*.

[34] Kunchithapautham K., Padmavathi B., Narayanan R. B., Kaliraj P., Scott A. L. (2003). Thioredoxin from *Brugia malayi*: defining a 16-kilodalton class of thioredoxins from nematodes. *Infection and Immunity*.

